# Gold nanoparticles embedded in a polymer as a 3D-printable dichroic nanocomposite material

**DOI:** 10.3762/bjnano.10.43

**Published:** 2019-02-12

**Authors:** Lars Kool, Anton Bunschoten, Aldrik H Velders, Vittorio Saggiomo

**Affiliations:** 1 Laboratory of BioNanoTechnology, Wageningen University and Research, P.O. Box 8038, 6700, EK Wageningen, The Netherlands

**Keywords:** 3D printing, dichroism, gold nanoparticles, nanocomposite

## Abstract

**Background:** Nanotechnology, even if unknowingly, has been used for millennia. The occurrence of shiny colors in pottery and glass made hundreds and thousand of years ago is due to the presence of nanoparticles in the fabrication of such ornaments. In the last decade, 3D printing has revolutionized fabrication and manufacturing processes, making it easier to produce, in a simple and fast way, 3D objects.

**Results:** In this paper we show how to fabricate a 3D-printable nanocomposite composed of dichroic gold nanoparticles and a 3D-printable polymer. The minute amount of gold nanoparticles used for obtaining the dichroic effect does not influence the mechanical properties of the polymer nor its printability. Thus, the nanocomposite can be easily 3D-printed using a standard 3D printer and shows a purple color in transmission and a brownish color in reflection.

**Conclusion:** This methodology can be used not only by artists, but also for studying the optical properties of nanoparticles or, for example, for the 3D fabrication of optical filters.

## Introduction

As evidenced by paleolithic cave paintings [1], humans have always been fascinated by colors. Next to the traditional inorganic and organic colorants, nanoparticles received the lion’s share, conferring shiny colors to pottery and glass in different eras. Copper nanoparticles, for example, have been found in red glass from the late Bronze Age, 1200–1000 BCE [2]. The use of nanoparticles as a colorant boomed around the 4th century CE within the Roman empire, where craftsmen, unaware of the existence of surface plasmon resonance [3], used metallic nanoparticles for coloring mosaic tiles, pottery and glass [4–5].

Metallic nanoparticles were also used for staining glass during medieval times, examples of which can still be found in many churches and cathedrals in Europe. The methodology for producing stained glass became reproducible in 1680, when the method for producing the “ruby red glass” was born [6]. The organic colorant of paintings from centuries ago is fading over time. However, pottery and glass embedded with nanoparticles made a thousand years ago are still the same shiny color as they were on the day of their production. Nanoparticles, in fact, are not subject to photobleaching, therefore, if the nanoparticles are stable over time, the color will persist for a longer time with respect to organic colorants.

A single glass piece from the 4th century puzzled scientists for long time: The Lycurgus cup [7]. This cup has a very peculiar dichroic filter property [8], as its color is dependent on the illumination angle, changing from clear red (transmittance) to opaque green (reflectance). It was later found that this optical property was due to gold nanoparticles (AuNPs) and silver nanoparticles (AgNPs) of different sizes and shapes [9–10]. However, only the Lycurgus cup, now stored in the British Museum, and six other broken pieces showing the same dichroic effect were found worldwide, hinting that the achievement of such an optical effect was most probably due to serendipity rather than to master craftsmanship.

In recent years, 3D printing technology has revolutionized the prototyping and fabrication process, pushing the manufacturing of objects from factories to houses. Within the 3D printing world, scientists have also started modifying 3D-printable plastics with, for example, catalysts [11] or TiO_2_ nanoparticles [12] to obtain new improved materials with special characteristics.

In this paper, we show how to fabricate a 3D-printable dichroic material using gold nanoparticles, jumping from the 4th century Roman glassmiths’ methods to a modern and widely spread technology.

## Results and Discussion

**Figure 1:**
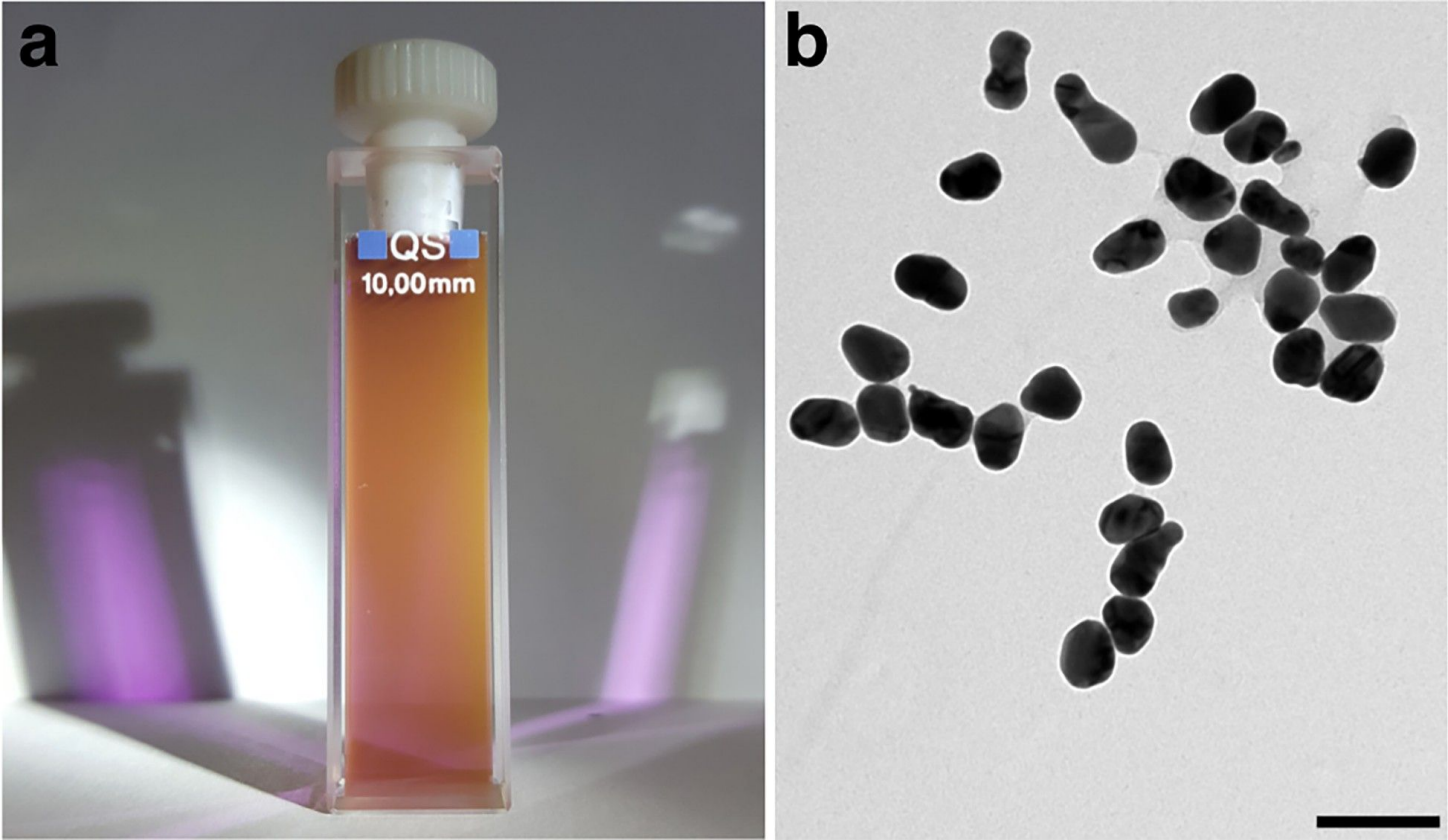
a) A dichroic AuNP solution. Here both the brown reflection and purple transmission can be observed. b) TEM micrograph of the AuNPs, scale bar 100 nm.

Dichroic AuNPs were prepared using a modified Turkevich method [13], thus reducing gold ions to gold nanoparticles using citrate as both reducing and capping agent. In the classical Turkevich method, a boiling chloroauric acid solution is reacted with citrate using a citrate/gold molar ratio of 10, producing AuNPs of around 10 nm. When this ratio is changed, the size of the obtained nanoparticles changes as well [14]. We discovered that a citrate/gold ratio between 0.6 and 0.8 produced dichroic nanoparticles that showed a brownish reflection and a purple transmission (Figure 1a). The nanoparticle solution was studied by transmission electron microscopy (TEM), showing that it was composed of polydisperse elongated nanoparticles of 50–60 nm with a mean aspect ratio of 1.4 (Figure 1b and Supporting Information File 1, Figure S1). In addition to the surface plasmon resonance color [15], the large size of the nanoparticles increases the Mie scattering [16], giving rise to the opaque reflection. However, the elongated shape of the nanoparticles may also contribute to the dichroism, as nanoparticles with an aspect ratio larger than 1.2 have been shown to possess dichroic properties [17]. Even a bimodal size distribution of spherical nanoparticles may present dichroism as well [18].

The presented synthesis is easy and fast, as it takes only few minutes to obtain the dichroic solution after the addition of the citrate. During the synthesis, the solution changed color multiple times: the yellow solution of the gold ions became blue one minute after the addition of the citrate solution. Two minutes later, the solution showed an intense black color, before becoming dichroic after another two minutes of boiling. The color change during the synthesis hints that the dichroic nanoparticle formation is not just seeded growth, but a more complex mechanism. Therefore, we studied the evolution of the nanoparticle formation over time using UV–vis spectroscopy and TEM (Supporting Information File 1, Figures S3 and S4). The time dependent study shows the formation of small gold nuclei that in time cluster together forming nanowire-like structures concomitant to the first color change. The second change of color, from ink-black to purple, is accompanied by an enhancement of the scattering, giving the purple solution a brown reflection. The explanation is that, while boiling, the gold nanowires fragment, creating nanoparticles with a large head and a slim and long tail, comparable to a tadpole. Over time the tail starts to shrink, due to intra-particle Ostwald ripening to minimize the total surface energy of the nanoparticle, yielding large ellipsoidal dichroic gold nanoparticles. Both steps, namely chain growth and interparticle ripening, have been previously reported in literature and are therefore not studied in depth here [19–20].

The next step was to embed the dichroic AuNP in a 3D-printable material, to be used with a standard off-the-shelf fused deposition modeling (FDM) 3D printer. We chose to use polyvinyl alcohol (PVA) as the nanoparticle carrier, because it is one of the most used 3D printing materials, it is water soluble, thus mixable with the AuNPs without the need to change solvents, and because it is known that PVA can be used as a capping agent for nanoparticles [21].

**Figure 2:**
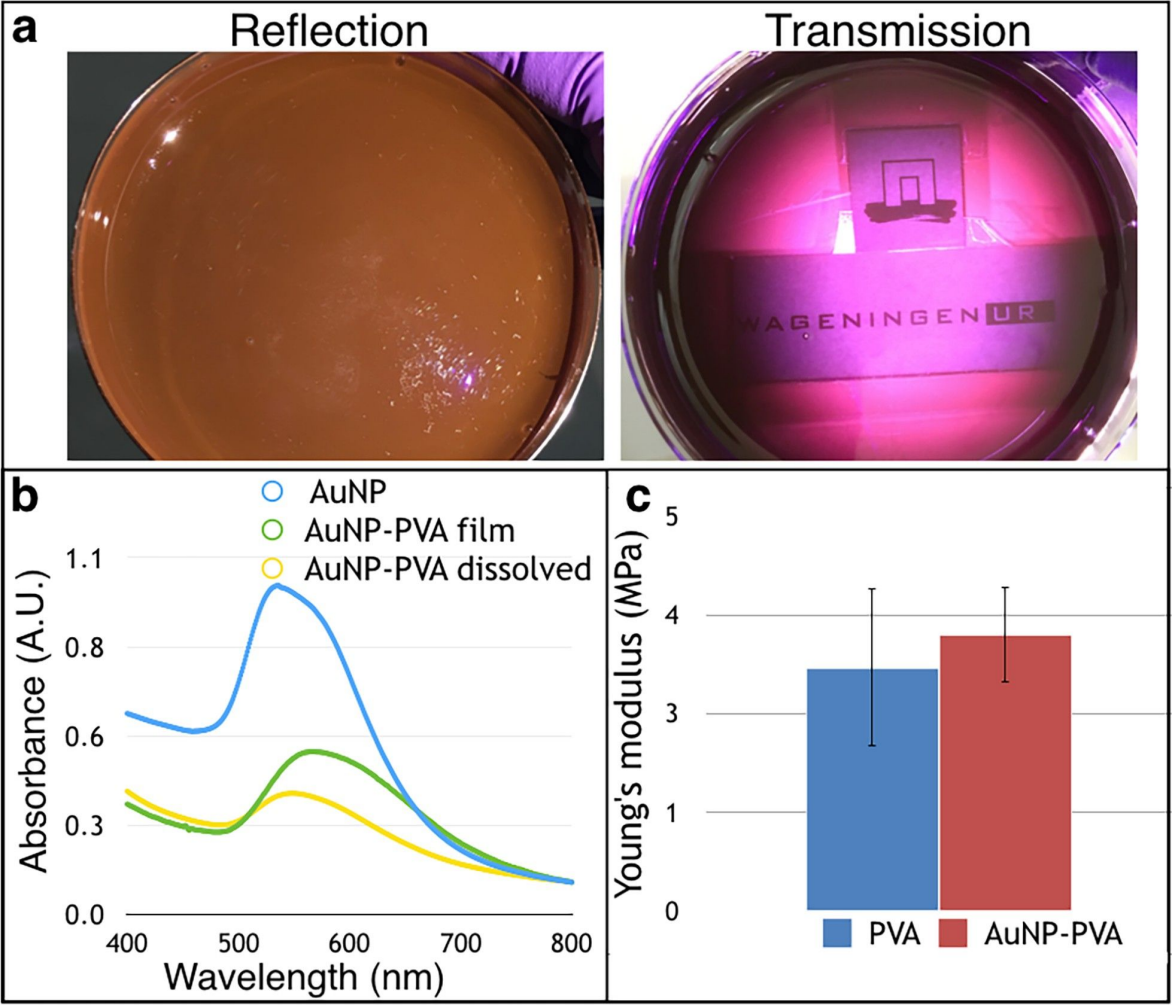
a) AuNP–PVA shows an opaque brown color in reflection and transparent purple in transmission. The transparency effect allows the text behind the plastic to be read. b) UV–vis spectra of the dichroic AuNPs (blue) and AuNP–PVA film (green) which shows a red shift probably due to a lack of solvent. When the AuNP–PVA film is dissolved in water, the AuNPs show again the same characteristic plasmon resonance band (yellow). c) Young’s modulus of the AuNP–PVA compared to pure PVA.

3D-printable grade PVA was then added to the dichroic solution to reach a concentration of 0.1% (w/w) of AuHCl_4_ (0.07% of gold in weight) in PVA. The mixture was left at 70 °C in a ventilated oven until it was dry, yielding the AuNP–PVA nanocomposite. The so formed AuNP–PVA shows the same dichroic effect of the solution – a brown opaque reflection and purple transparent transmission (Figure 2a, Supporting Information File 2). Standard gold nanoparticles of less than 20 nm embedded in PVA do not show this dichroic property, as they give a red transparent color to the PVA, here named “ruby plastic” as reference to the first reproducible nanoparticle embedded glass “ruby glass” (Supporting Information File 1, Figure S8).

The surface plasmon resonance band of the gold nanoparticles in the PVA film shows a redshift of 20 nm with respect to the original dichroic solution (Figure 2b). As the concentration of gold atoms in the PVA is only 0.07%, this shift cannot be attributed to the plasmon–plasmon coupling between nanoparticles, but it is probably due to the difference in the interaction between solvent and nanoparticles in water and when embedded in solid PVA. This effect is proven when the AuNP–PVA is dissolved in water, releasing the nanoparticles, giving almost the same absorption band as the original dichroic solution (Figure 2b and Supporting Information File 1, Figure S6). The small difference in absorbance of 6 nm can be attributed to the capping ligand on the gold nanoparticles: citrate in the original dichroic solution and PVA in the AuNP–PVA dissolved in water.

We compared the TEM results of the original dichroic solution to the AuNP–PVA dissolved in water (Supporting Information File 1, Figure S5) to find that the nanoparticles were still of the same size and shape as the original ones, showing that embedding in PVA does not influence the stability of the nanoparticles.

Lastly, we extruded the AuNP–PVA to fabricate a filament for FDM printing. As the percentage of AuHCl_4_ is only 0.1% with respect to PVA, we did not envision a drastic change in the mechanical properties between pure PVA and AuNP–PVA. To test this, we 3D-printed dog-bone-shaped strips of plastic (2.5 × 0.4 × 0.1 cm) and tested the elastic modulus (Young’s modulus) using dynamic mechanical analysis (DMA). The pure PVA and the AuNP–PVA, as expected, did not show significant differences in the elastic modulus giving an average of 3.5 ± 0.8 MPa and 3.8 ± 0.6 MPa, respectively (Figure 2c). The relatively high standard deviation error is given by the intrinsic layer-by-layer 3D fabrication method.

**Figure 3:**
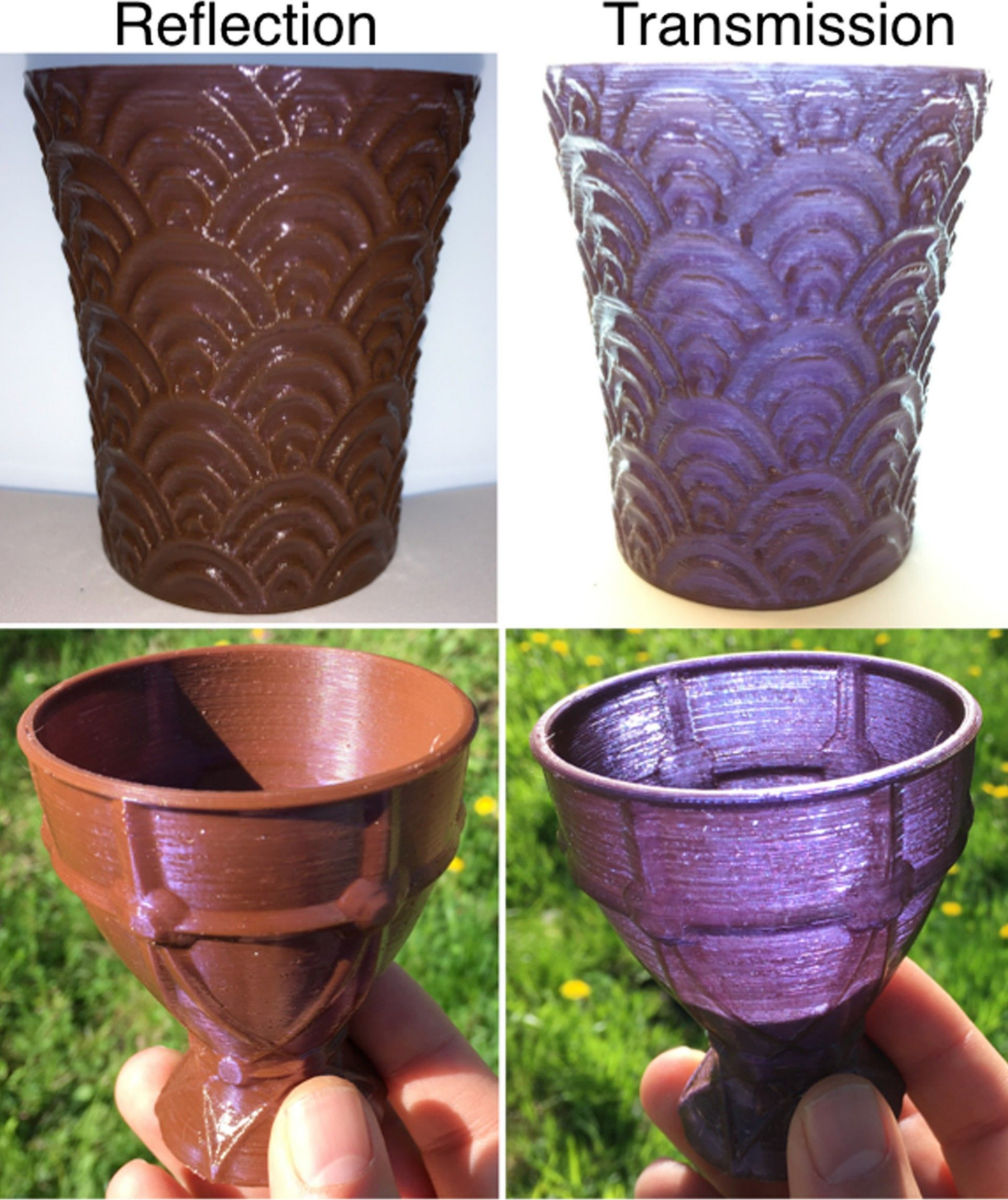
3D-printed cups using AuNP–PVA material showing the dichroic effect in artificial and sunlight.

The filament was used for printing different “21st century Lycurgus cups” (Figure 3, Supporting Information File 1, Figure S9–11 and Supporting Information File 3). The small percentage of gold in the filament, also in this case, did not influence the printability of the plastic, and the same parameters for printing pure PVA were successfully used for printing AuNP–PVA. At the used percentage of gold in PVA, the dichroic effect in the printed objects can be detected when the wall thickness of the 3D-printed object is larger than 0.4 mm. The 3D-printed parts can also be smoothened by quickly washing the parts in water (Supporting Information File 1, Figure S12). The water solubility of PVA has, however, pros and cons: Due to its ease of printability and water solubility, PVA is the most used supporting material. On the other hand, the water solubility of PVA is a problem when a strong resistant material is needed. For example one could not use the cup for drinking water as it will dissolve after a few minutes. To overcome this problem, we coated the 3D-printed cup with a layer of polydimethylsiloxane (PDMS), a flexible, nontoxic and food-safe transparent elastomer [22]. The cup was brushed with liquid PDMS and it was cured at 70 °C for a few hours. The PDMS-coated AuNP–PVA cup was able to withstand water without any leakage (Supporting Information File 1, Figure S13 and Supporting Information File 4). The cups, both coated and non-coated, were stable at room temperature and ambient light for at least six months, without changing the dichroic effect.

## Conclusion

In conclusion, we showed how to synthesize and embed dichroic nanoparticles in 3D-printable material. The AuNP–PVA nanocomposite is mechanically similar to the bare plastic material, and its dichroic optical properties are similar to the the AuNP solution. The 3D-printed objects can be coated to achieve water impermeability and stability at room temperature for long time. We can envision this methodology to be used not only by artists, but also for studying optical properties of nanoparticles or, for example, for the 3D fabrication of optical filters.

## Experimental

### General

Chloroauric acid trihydrate and trisodium citrate dihydrate were obtained from Alfa Aesar and used without further purification. The PVA 3 mm filament (Ultimaker) was obtained from Makerpoint. A Shimadzu UV1601 UV–vis spectrometer was used for the UV–vis study. The samples were imaged in a JEOL 1400Plus TEM operating at 120 kV. The mean aspect ratio of the AuNPs was calculated by measuring approximately 800 particles from 24 micrographs. A TE instruments DMA Q800 instrument was used for the mechanical analysis of the plastics using the stress/strain program with a force ramp rate of 1 N/min at isothermal (25 °C) temperature. Four samples of the pure PVA and four samples of the AuNP–PVA materials were tested, where each sample was also flipped upside down and the stress/strain was recorded again. The average of the measurements was used in Figure 2c with the standard deviation shown as the error bar.

### Synthesis of dichroic gold nanoparticles

0.5 mL of a 34 mM citrate solution in distilled water was added, in one shot, to a 100 mL boiling solution of 0.25 mM HAuCl_4_ in distilled water under vigorous stirring. The solution was left boiling while stirring for 5 min, until a brown reflection could be seen. The solution was then passively cooled to room temperature leaving the solution on the bench and stored for further use. The solution can also be cooled to 50 °C to directly continue with the AuNP–PVA fabrication process.

### Fabrication of AuNP–PVA nanocomposite

Small pieces of PVA 3D-printable filament were added to the dichroic solution at 50 °C to reach a final concentration of 0.1% (w/w) of AuHCl_4_ in PVA (8.5 g PVA per 100 mL of 0.25 mM HAuCl_4_ solution), taking in account that the maximum solubility of PVA in water is ≈20% (w/w). A temperature below 50 °C produces less foam while gently stirring the PVA and it is, in general, easier to handle. When all the PVA is dissolved, the AuNP–PVA solution was transferred to plastic petri dishes and left in a ventilated oven at 70 °C until all the water was evaporated (depending by the volume, surface area and ventilation, this process takes between 12 and 24 h), yielding the hard AuNP–PVA plastic. Plastic petri dishes should be used instead of glass, as the PVA sticks to the glass like glue, making the unmolding more difficult. The plastic was shredded and extruded using a Thermo Fisher PolyLab OS single screw extruder at 180 °C with a pulling speed of 210 mm/min to a 3 mm wire for the successive printing.

### 3D printing

The 3D designs of the cups were downloaded from thingiverse.com: “Weekly cup nr1” by joris https://www.thingiverse.com/thing:40770, “Weekly cup 32” by joris https://www.thingiverse.com/thing:134879, “Goblet, Grail, Chalice or ornate Vase” by idig3d https://www.thingiverse.com/thing:1907150, and used under the creative commons CC-BY-NC 3.0 and CC-BY-NC-SA 3.0 licenses.

The 3D designs were sliced with Cura 3.1.0 and printed on an Ultimaker 2+ FDM printer using a 0.4 mm nozzle (0.2 mm layer height and 0.8 mm wall thickness) and a 0.6 mm nozzle (0.3 mm height and 0.6 mm wall thickness). The following printing parameters were used: printing temperature 215 °C, build plate temperature 60 °C, fan speed 50%, print speed 50 mm/s.

The printed parts were smoothened by brushing the prints with a small wet brush. PDMS-coated cups were made by mixing and stirring 10:1 Sylgard 184:curing agent. This solution was brushed on top of the cup and it was left standing at room temperature for 12 h. Another coating of PDMS was done in the same way and then the cup was left in an oven at 70 °C for 2 h.

## Supporting Information

File 1 Supporting information for aspect ratio of AuNPs, UV–vis and TEM analyses of AuNPs and AuNP-PVA nanocomposites, pictures of AuNP-PVA 3D-printed cups and their coating.

File 2 AuNP-PVA.
Video showing the dichroic AuNPs embedded in PVA.

File 3 3D-printed AuNP-PVA.
Video showing the dichroic 3D-printed AuNPs embedded in PVA.

File 4 3D-printed and coated AuNP-PVA.
Video showing the coated dichroic AuNPs embedded in PVA and their water stability.
