# Supplementary material for: Gold nanoparticles embedded in a polymer as a 3D-printable dichroic nanocomposite material
Source: Beilstein J Nanotechnol. 2019 Feb 12;10:442–7. doi: 10.3762/bjnano.10.43 (PMC6404512; doi:10.3762/bjnano.10.43)
Supplement: File 1 — Supporting information for aspect ratio of AuNPs, UV–vis and TEM analyses of AuNPs and AuNP-PVA nanocomposites, pictures of AuNP-PVA 3D-printed cups and their coating. [file Beilstein_J_Nanotechnol-10-442-s001.pdf]

BEILSTEIN JOURNAL OF NANOTECHNOLOGY

Supporting Information

for

Gold nanoparticles embedded in a polymer as a 3D-printable dichroic nanocomposite material

Lars Kool, Anton Bunschoten, Aldrik H. Velders and Vittorio Saggiomo

Beilstein J. Nanotechnol. **2019,** *10,* 442–447. doi:10.3762/bjnano.10.43

Supporting information for aspect ratio of AuNPs, UV–vis and TEM analyses of AuNPs and AuNP-PVA nanocomposites, pictures of AuNP-PVA 3D-printed cups and their coating

License and Terms: This is a supporting information file under the terms of the Creative Commons Attribution License (http://creativecommons.org/licenses/by/4.0). Please note that the reuse, redistribution and reproduction in particular requires that the authors and source are credited.
The license is subject to the *Beilstein Journal of Nanotechnology* terms and conditions: (https://www.beilstein-journals.org/bjnano)

Aspect ratio of AuNP	S1
Analysis of the synthesis via:	
UV-Vis	S2
	S3
TEM	S4
Study of films and dissolved AuNP-PVA	S5
UV-Vis	S6
TEM	S7
Small AuNP embedded in PVA	S8
3D printed dichroic AuNP-PVA cups	S9
	S10
	S11
Smoothened and coated AuNP-PVA	S12
Watertight AuNP-PVA 3D printed cup	S13

Supporting Information File 2, Video S1: dichroic effect of AuNP-PVA
Supporting Information File 3, Video S2: 3D printed AuNP-PVA nanocomposite
Supporting Information File 4, Video S3: Watertight AuNP-PVA 3D printed cup

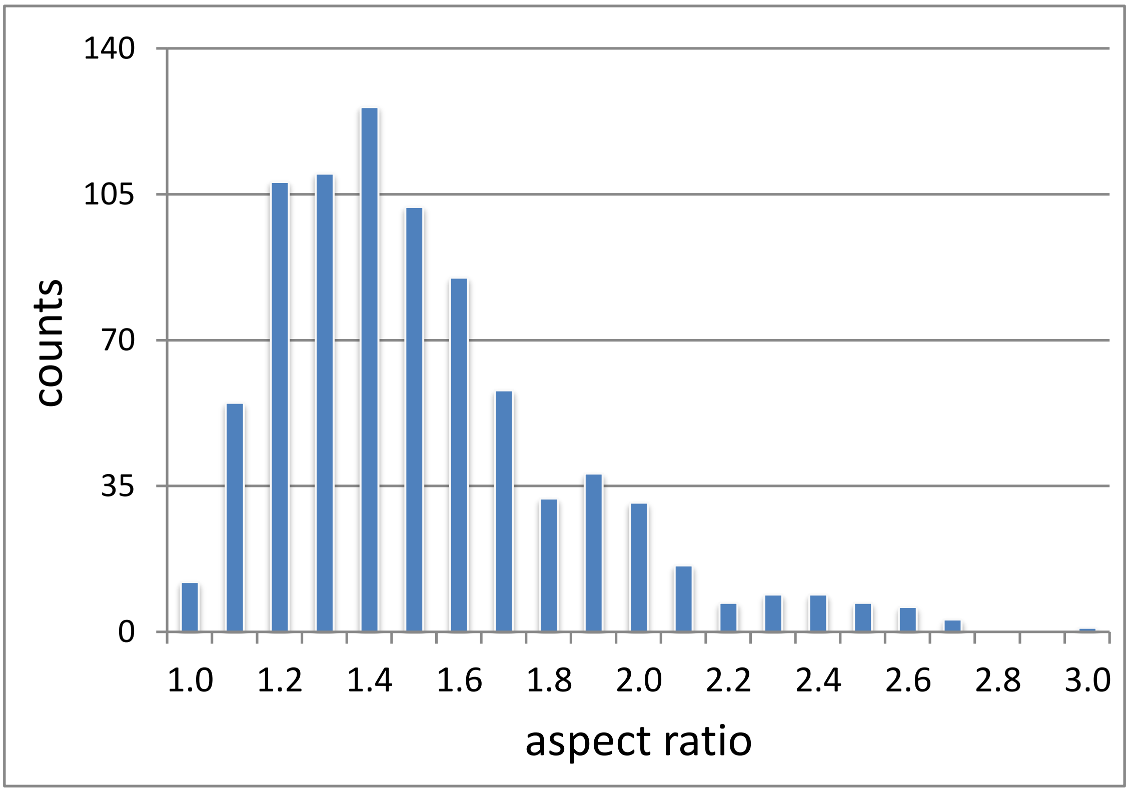

Figure S1: Aspect ratio of the synthesized AuNP.

Analysis of the synthesis

During a synthesis, ~1.5 mL samples were taken every 30 seconds for 4 minutes and samples were taken after 5 and 10 minutes (Figure S1). Directly after taking a sample, it was cooled in an ice-bath to quench the reaction. The samples were then examined by UV-Vis and TEM.

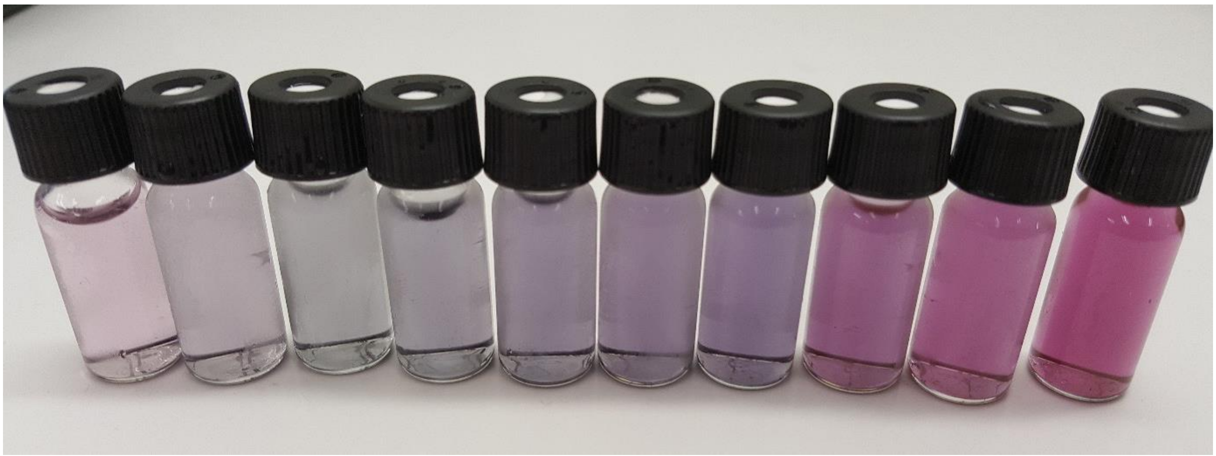

Figure S2: Samples taken during the synthesis. The samples were taken at a 30 second interval, with exception of the last 2. They were taken at 5 and 10 minutes from the start of the synthesis. Only the last 3 samples display the dichroic effect (not visible on this photo due to the shorter path length).

UV-Vis analysis:

The visual color changes in Figure S1 was analyzed by UV-Vis. During boiling, the absorbance gradually increases. Which corresponds to the increase in color observed during the synthesis. The color also changes during the synthesis, from grey (equal absorbance across the whole spectrum) to purple violet (absorbance peak at 530-540 nm). Further boiling sharpens the SPR peak, indicating intra-particle ripening even after dichroic nanoparticles have formed.

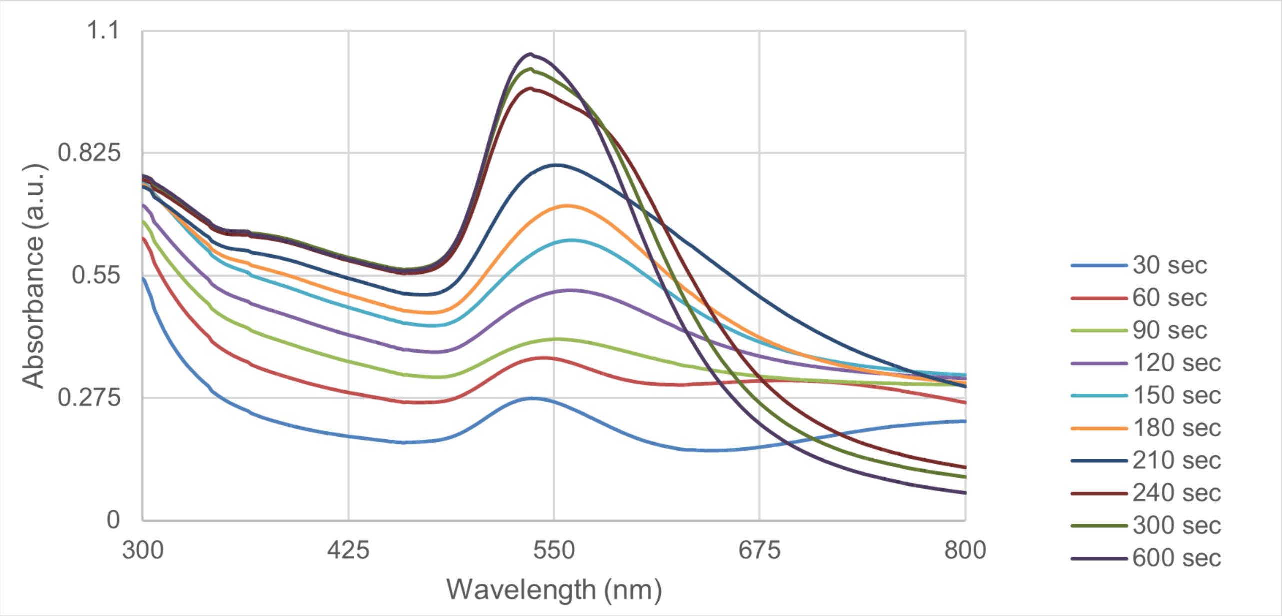

Figure S3: Absorbance of the gold nanoparticle reaction mixture over time. The last sample is corrected for the evaporation during the synthesis.

TEM analysis:

The samples taken over time were also analyzed by transmission electron microscopy (TEM). Here the transitions were harder to discriminate and only 3 major stages could be discriminated: the formation of seeds and the aggregation of seeds into small networks (Figure S4 a), presence of nanoparticle networks (from ~100 nm to ~1000 nm diameter) (Figure S4 b) and the presence of non-spherical nanoparticles (Figure S4 c,d).

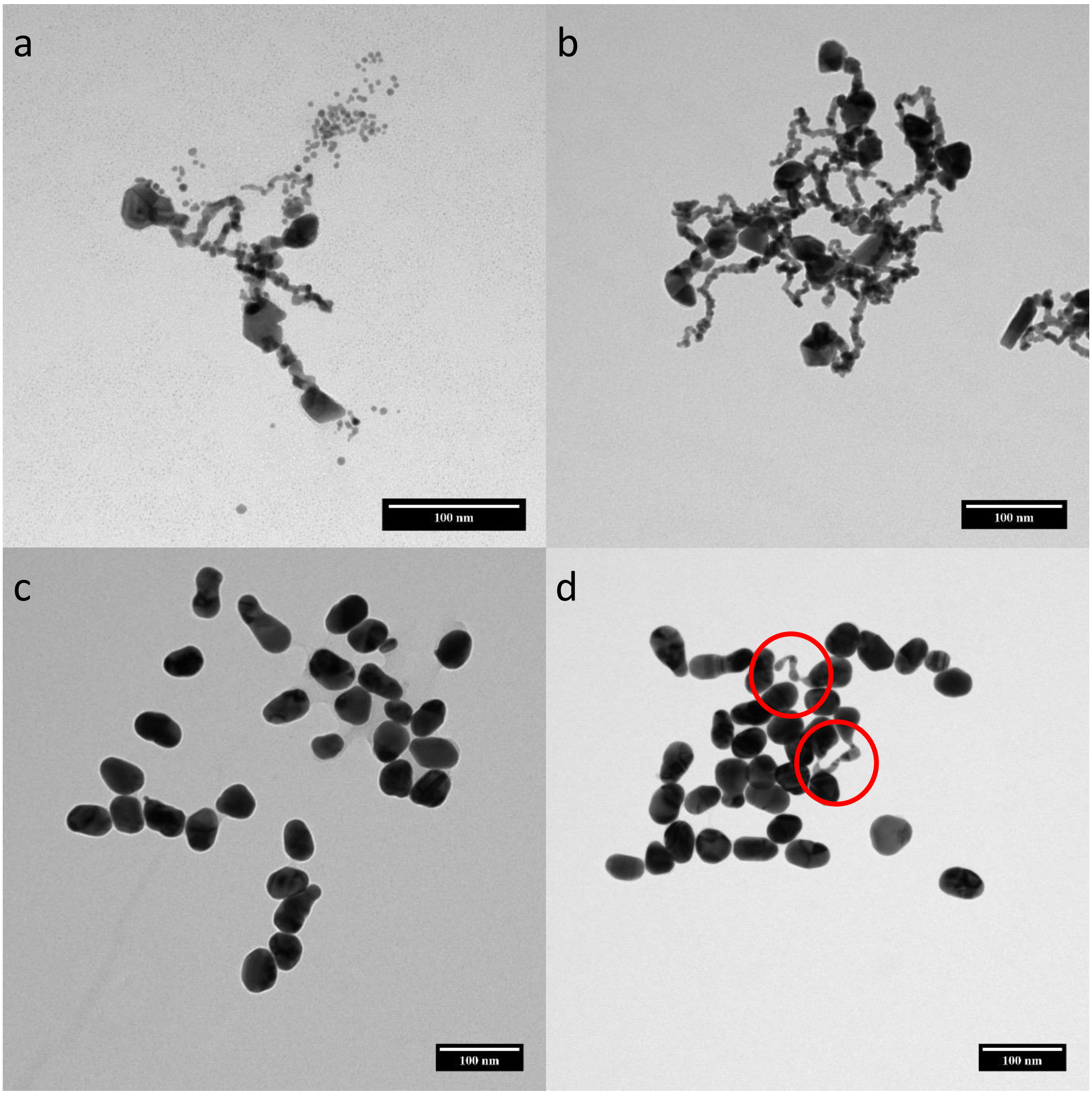

Figure S4: TEM images of the gold nanoparticle solution after a) 30 seconds, b) 1.5 minutes, c) 3 minutes, and d) 5 minutes. After 5 minutes not all nanoparticles are ripened, as some still have a small tail (highlighted in red circles). Scale bar = 100nm.

Study of films and dissolved AuNP-PVA:

A thin film of AuNP-PVA was made to study the characteristics of the AuNP embedded in PVA (Figure S5 a). The AuNP-PVA was also dissolved back in water to study the AuNP using UV-Vis and TEM (Figure S5 b,c).

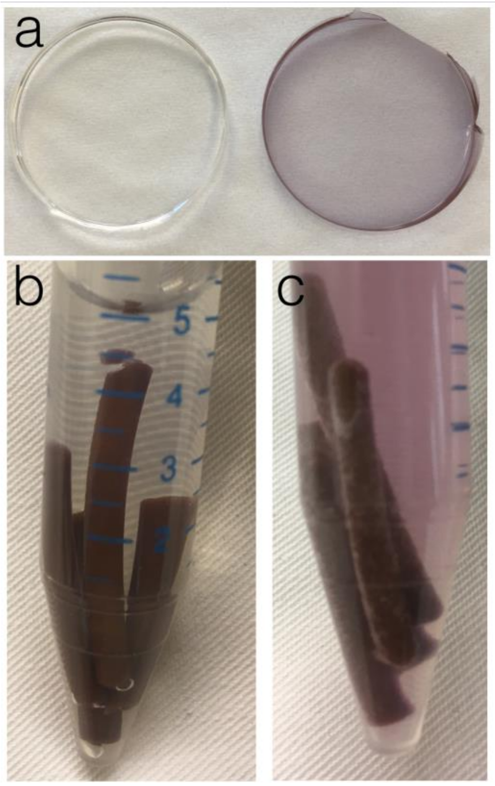

Figure S5: a) Thin films of PVA on the left and AuNP-PVA on the right for the UV-Vis study. The AuNP-PVA can be b) dissolved back in water and, c) after 10 minutes shaking the plastic start dissolving, thus releasing the gold nanoparticles in solution.

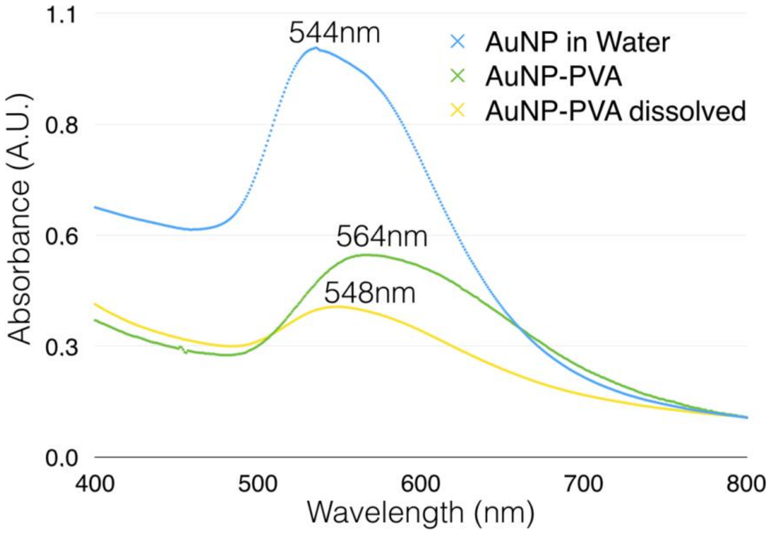

Figure S6: UV-Vis of the freshly synthesized dichroic AuNP (blue), the AuNP-PVA film from figure S5a (green) and after dissolving the AuNP-PVA in water from figure S5c (yellow).

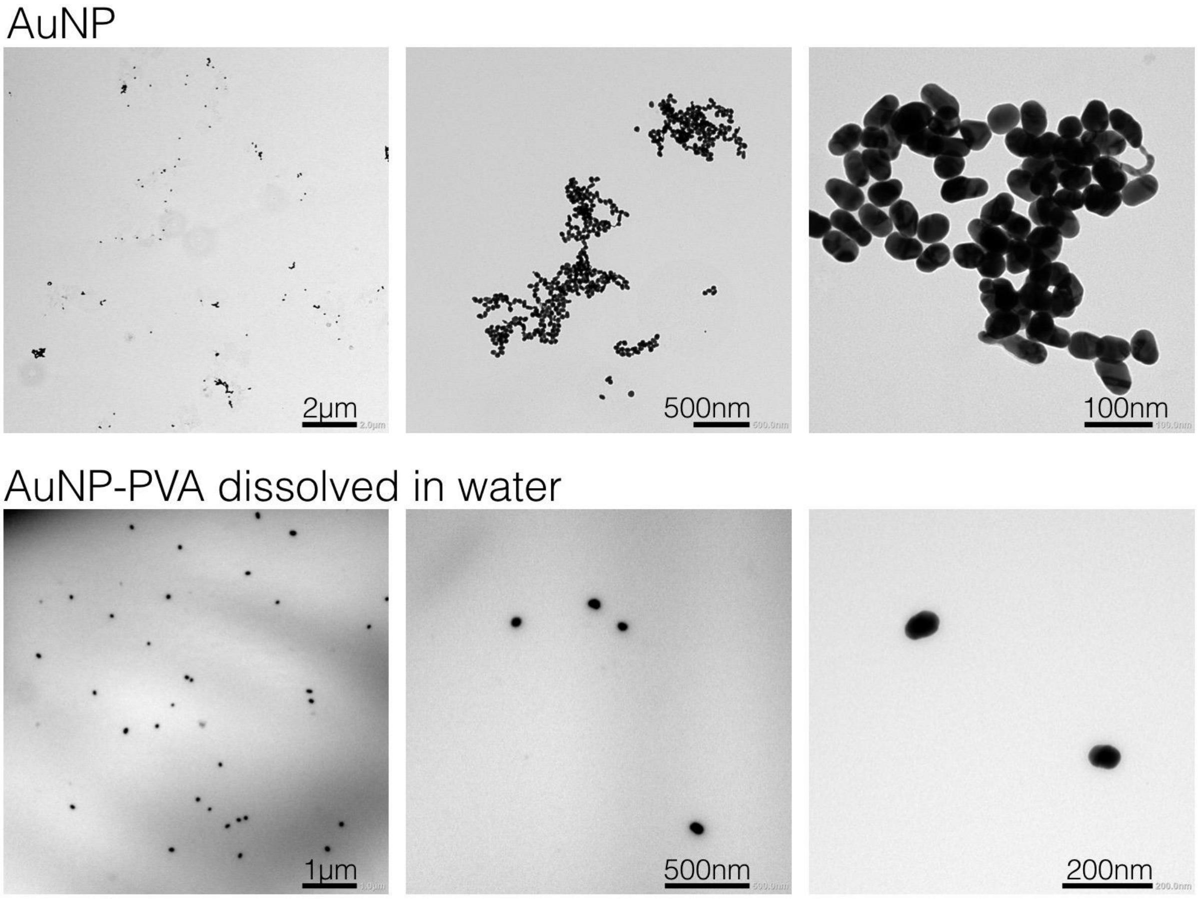

Figure S7: TEM of the freshly synthesized AuNP on top, and TEM pictures of the AuNP-PVA dissolved in water (from Figure S5c).

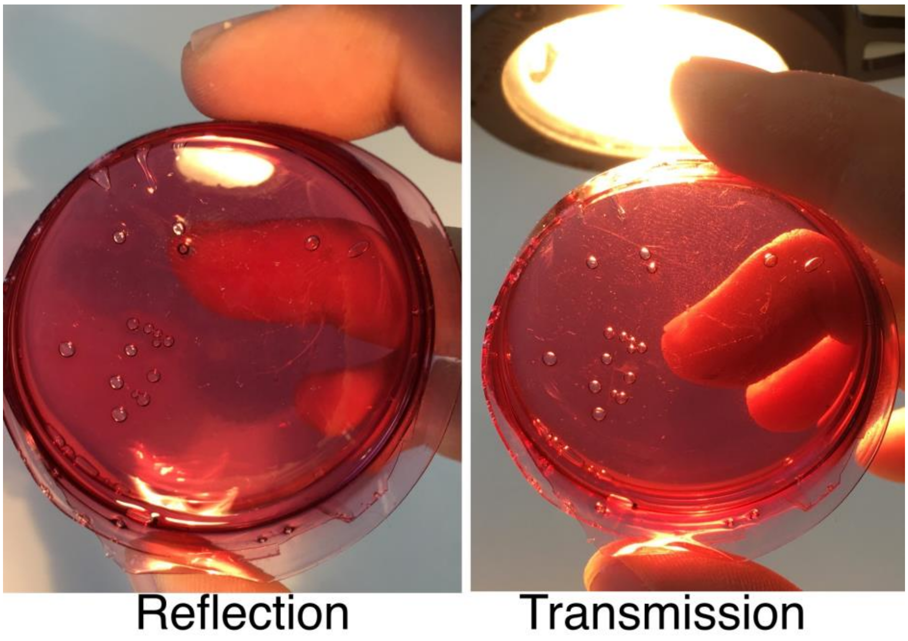

Figure S8: Small (<20nm) AuNP embedded in PVA. Smaller nanoparticles do not show dichroism when embedded in PVA.

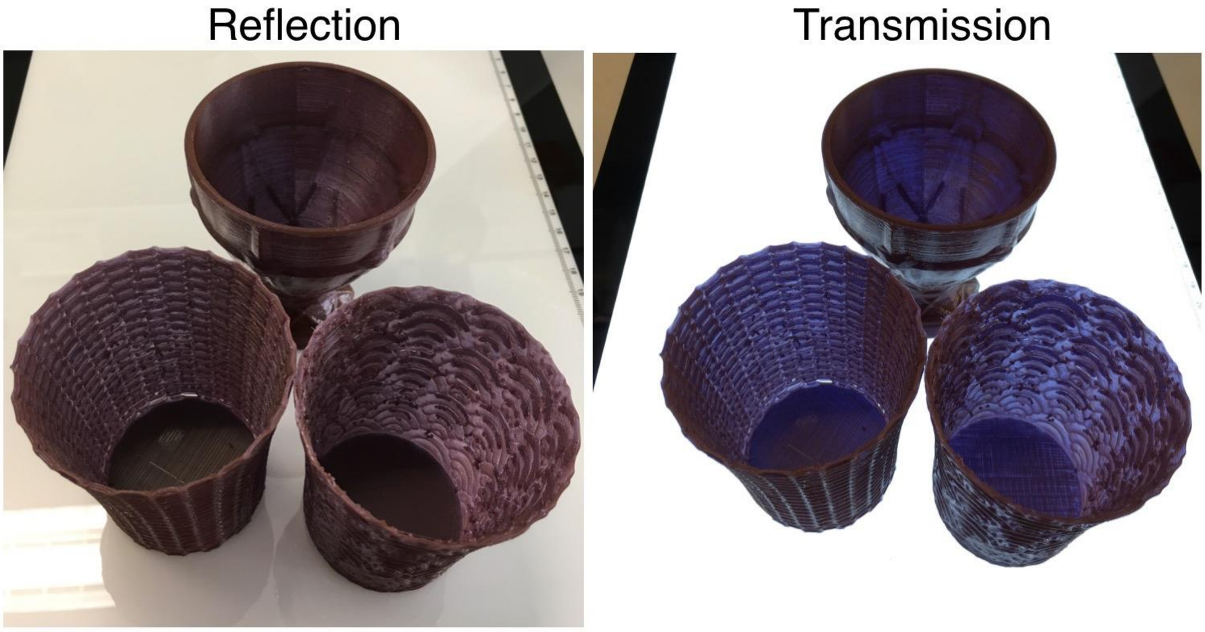

Figure S9: 3D printed dichroic AuNP-PVA cups.

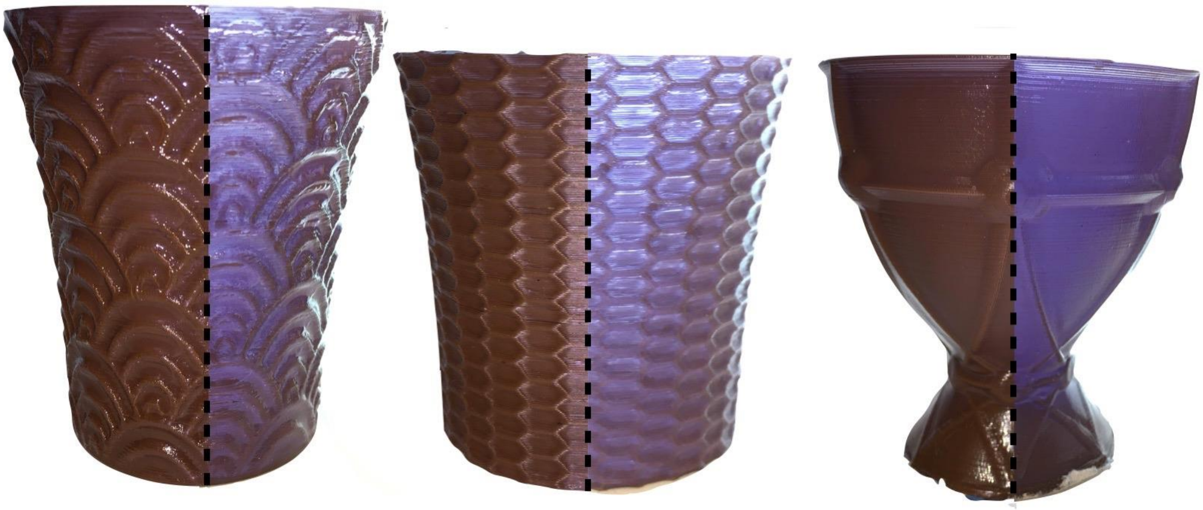

Figure S10: Overlay of the 3D printed dichroic AuNP-PVA cups, half reflection on the left and half transmission on the right.

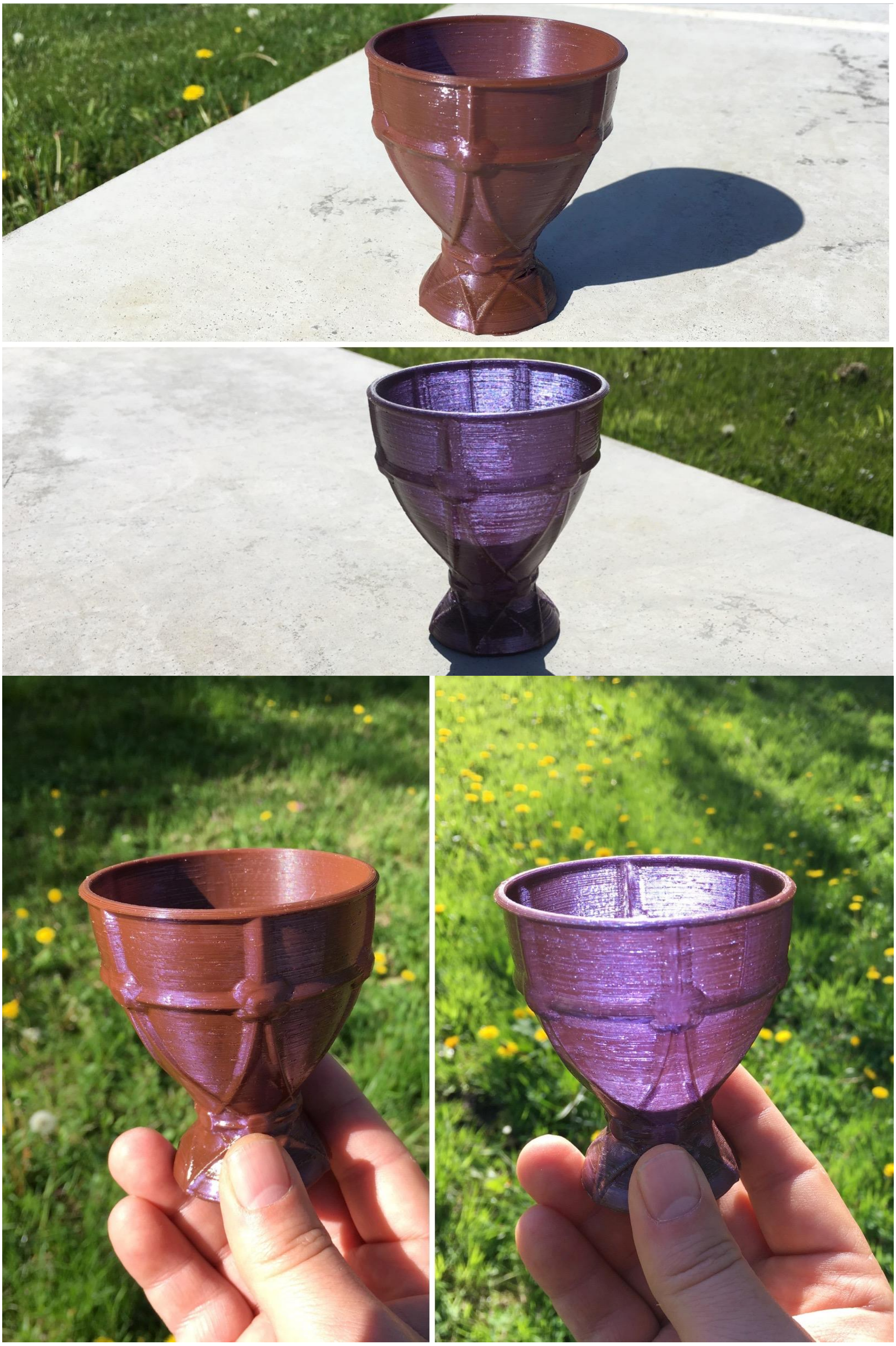

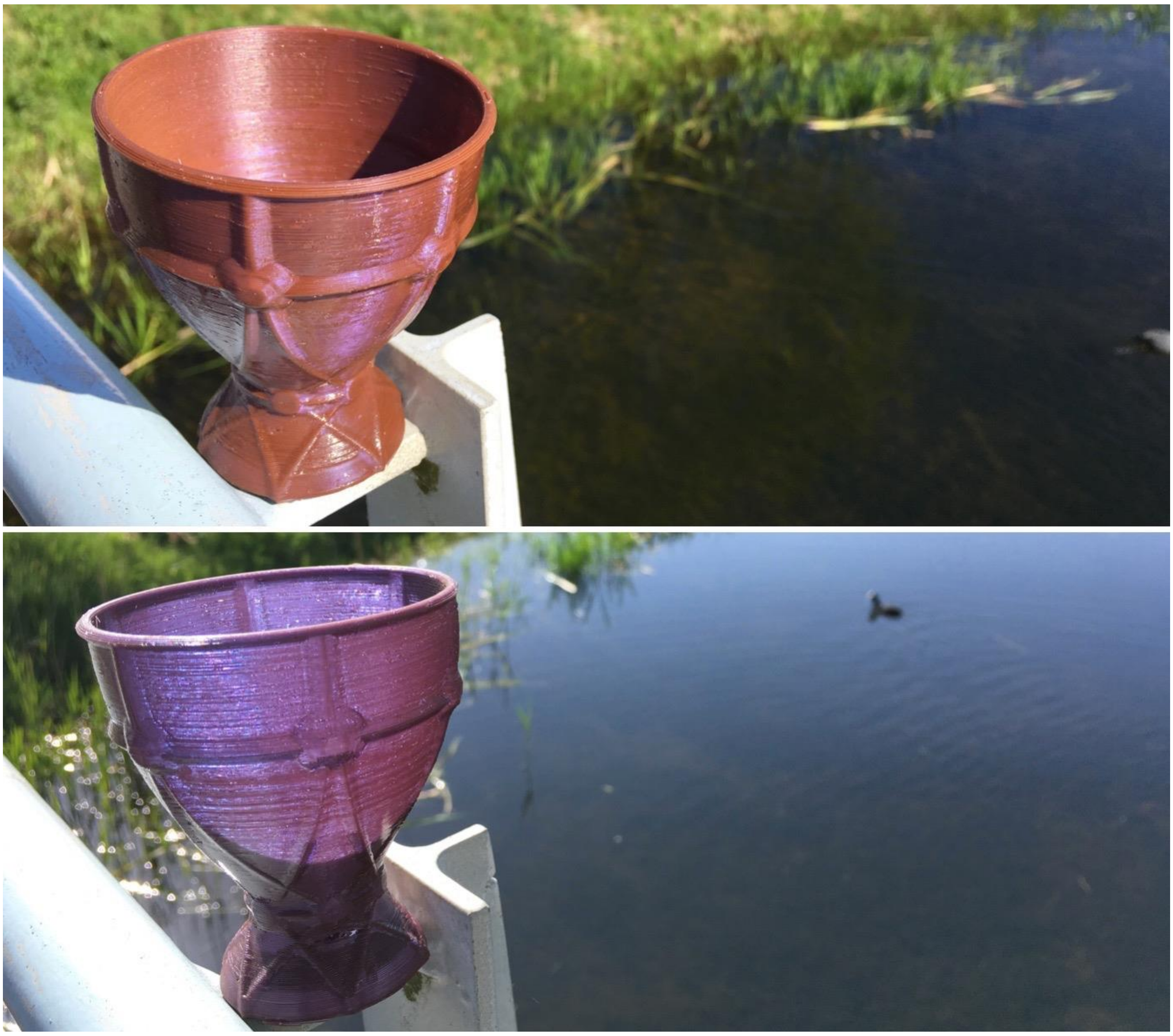

Figure S11: 3D printed dichroic AuNP-PVA cups under natural illumination.

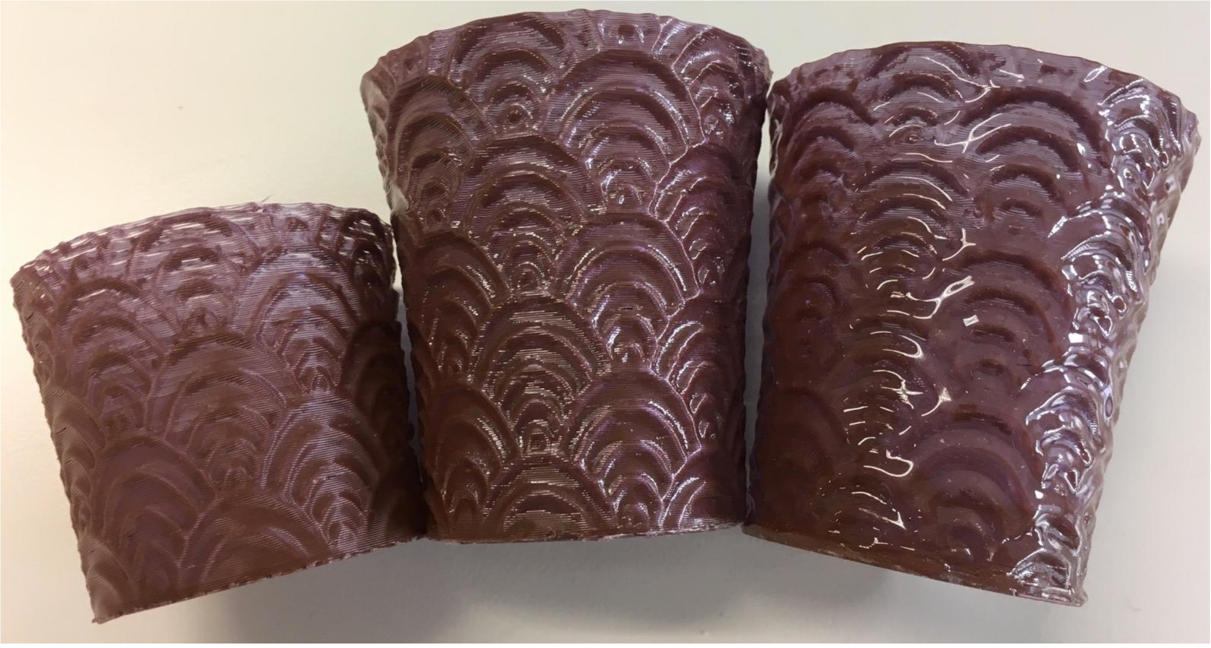

Figure S12: From left to right: pristine 3D printed AuNP-PVA cup, water smoothened cup and, on the right, a PDMS coated one.

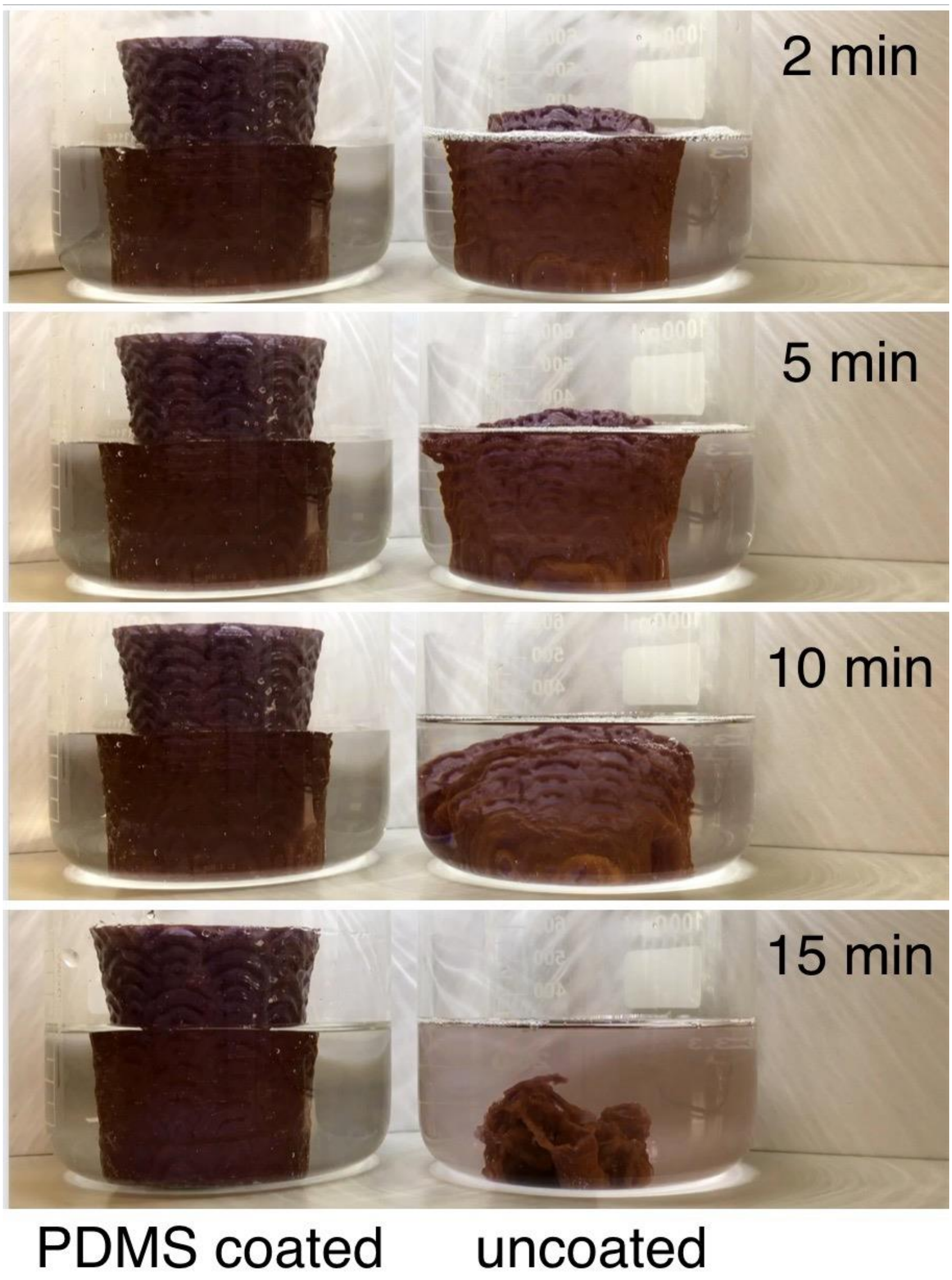

Figure S13: The PDMS coated 3D printed AuNP-PVA cup is both rather tight and water resistant. The pristine AuNP-PVA cup is dissolvable in water.
